# Supplementary material for: Metataxonomic Analysis Demonstrates a Shift in Duodenal Microbiota in Patients with Obstructive Jaundice
Source: Microorganisms. 2023 Jun 18;11(6):1611. doi: 10.3390/microorganisms11061611 (PMC10301301; doi:10.3390/microorganisms11061611)
Supplement: Supplementary file 1 [file microorganisms-11-01611-s001.zip › Supplementary Tables.pdf]

SUPPLEMENTARY TABLES

Table S1. Diversity indices of individual samples in the jaundice group.

	Observed	Chao1	se,chao1	ACE	se,ACE	Shannon	Simpson	InvSimpson	Fisher
S020	75,00	75,00	0,00	75,00	2,81	2,13	0,75	4,02	8,84
S021	51,00	51,00	0,00	NA	NA	3,04	0,91	11,44	5,72
S022	35,00	35,00	0,00	NA	NA	2,63	0,88	8,40	3,75
S023	113,00	116,00	4,18	113,94	5,13	2,74	0,89	8,73	14,10
S024	62,00	62,00	0,00	62,00	0,99	2,57	0,80	4,96	7,13
S025	77,00	77,00	0,50	77,55	1,46	3,05	0,92	12,88	9,11
S026	61,00	61,00	0,00	61,00	1,69	2,96	0,90	10,49	7,00
S027	87,00	87,00	0,50	87,16	2,65	3,34	0,94	16,60	10,46
S028	87,00	87,00	0,00	87,00	2,84	2,48	0,75	4,02	10,46
S029	60,00	60,00	0,25	60,23	3,25	1,47	0,52	2,10	6,87
S030	73,00	74,00	2,33	74,00	2,34	2,49	0,82	5,57	8,57
S031	49,00	49,00	0,00	49,00	0,99	2,92	0,91	10,57	5,47
S032	65,00	65,00	0,00	65,00	0,99	2,66	0,82	5,65	7,52
S033	77,00	77,00	0,00	NA	NA	3,37	0,94	16,87	9,11
S034	62,00	62,00	0,00	NA	NA	2,73	0,83	5,75	7,13
S035	86,00	86,00	0,00	NA	NA	3,52	0,95	19,03	10,33
S036	79,00	79,00	0,00	79,00	2,16	2,23	0,80	4,91	9,38
S037	61,00	61,00	0,00	61,00	0,99	3,13	0,92	13,12	7,00
S038	78,00	78,00	0,00	78,00	1,40	2,32	0,74	3,82	9,24

Table S2. Mean distribution of phyla comparing control and jaundice cohorts.

Phyla	Control	Jaundice
Arthrobacter	6,07	4,94
Fusobacterium	1,19	4,03
Haemophilus	4,00	2,39
Helicobacter	6,23	2,36
Pelomonas	8,29	6,43
Prevotella	7,42	9,55
Propionibacterium	3,69	5,48
Pseudomonas	11,02	14,59
Ralstonia	3,31	2,13
Streptococcus	6,53	5,20
Veillonella	3,62	3,72
Other	38,64	39,19

Table S3. Mean distribution of genera comparing patients with cholelithiasis and HOP (head of pancreas) mass.

Genera	Cholelithiasis	HOP mass
Arthrobacter	5,45	4,40
Bergeyella	0	3,63
Haemophilus	4,09	1,03
Helicobacter	4,42	0,03

Microbacterium	1,38	3,78
Pelomonas	7,92	6,94
Prevotella	5,46	4,45
Propionibacterium	3,43	7,24
Pseudomonas	21,14	4,66
Ralstonia	2,69	1,91
Staphylococcus	0,78	3,21
Streptococcus	7,81	10,80
Veillonella	5,22	12,46
Other	23,43	26,04

Table S4. Relative abundances, expressed as a percentage, of the bacterial phyla in the duodenum of patients when grouped according to the presence or absence of stone disease.

Phyla	Stone disease	Non-stone disease
Actinobacteria	14,90	18,44
Bacillota	15,13	18,71
Bacteriodota	12,11	13,54
Chlamydiae	0	0
Cyanobacteria	0	0,02
Deferribacteres	0	0,02
Deinococcota	0,03	0,01
Fibrobacteres	0	0
Fusobacteria	2,89	2,43
Nitrospirae	0	0
Planctomycetes	0,02	0,01
Pseudomonadota	54,62	46,54
Spirochaetes	0,18	0,13
Synergistetes	0,05	0
Tenericutes	0,07	0,13
Verrucimicrobiota	0	0,01

Table S5. Relative abundances, expressed as a percentage, of the bacterial phyla in the duodenum of patients, comparing patients with benign and malignant conditions for jaundiced as well as control patients.

Phyla	Control	Malignant	Benign
Actinomycetota	16,79	20,21	13,83
Bacillota	16,41	24,25	12,84
Bacteriodota	11,00	14,07	14,76
Chlamydiae	0	0,01	0
Cyanobacteria	0,02	0	0
Deferribacteres	0,02	0	0
Deinococcota	0,02	0,01	0,03
Fibrobacteres	0,01	0	0

Fusobacteria	1,27	3,44	4,49
Nitrospirae	0	0	0
Planctomycetes	0	0,01	0,03
Proteobacteria	54,34	37,56	53,59
Spirochaetes	0,08	0,18	0,25
Synergistetes	0	0,003764	0,08
Tenericutes	0,03	0,260849	0,10
Verrucimicrobiota	0,01	0	0

Table S6. Bray-Curtis measure of dissimilarity of the beta-diversity of the microbiota of the duodenal mucosa factoring in multiple variables.

Groups	df	R^2	F-value	p-value
Control vs Jaundice	1	0,0225	0,8276	0,6224
Gender	1	0,02386	0,8801	0,5415
CLO test	1	0,02133	0,7845	0,6903
Underlying disease	8	0,22942	1,0793	0,3187
Benign vs Malignant	2	0,03852	0,7011	0,9091
Stone disease vs Non-Stone disease	1	0,03591	1,3408	0,1688
Control vs Jaundice x Gender	1	0,02528	0,9255	0,5405
Control vs Jaundice x CLO test	1	0,01973	0,7217	0,7323
Control vs Jaundice x Underlying disease	#	#	#	#
Control vs Jaundice x Benign vs Malignant	#	#	#	#
Control vs Jaundice x Stone disease vs Non-stone disease	1	0,01949	0,7163	0,7572
Underlying disease x Gender	3	0,0619	0,7774	0,81219
Underlying disease x CLO test	3	0,0744	0,9146	0,5624
Underlying disease x Benign vs Malignant	#	#	#	#
Underlying disease x Stone disease vs Non-stone disease	#	#	#	#
Benign vs Malignant x Gender	2	0,0464	0,8326	0,6883
Benign vs Malignant x CLO test	2	0,0565	1,0234	0,4186
Benign vs Malignant x Stone disease vs Non-stone disease	#	#	#	#
Stone disease vs Non-stone disease x Gender	1	0,01661	0,6126	0,8911
Stone disease vs Non-stone disease x CLO test	1	0,04852	1,8391	0,04895*
Gender x CLO test	1	0,01921	0,7028	0,7592
Age	1	0,01897	0,6963	0,7732
Control vs Jaundice x Age	1	0,02479	0,9055	0,5155
Gender x Age	1	0,0227	0,8258	0,6234
CLO test x Age	1	0,02425	0,8828	0,5445
Underlying disease x Age	3	0,05787	0,6893	0,8991

Benign vs Malignant x Age	2	0,0538	0,9686	0,4835
Stone disease vs Non-Stone disease x Age	1	0,02445	0,9007	0,5524

# - represents no valid result. * - significant difference (p-value < 0.05)

Table S7. Relative abundances, expressed as a percentage, of the bacterial phyla in the duodenum of patients, comparing patients based on the underlying conditions at the time of endoscopy.

Phyla	Choledocholithiasis	Cholelithiasis	Distal cholangiocarcinoma	Epigastric pain	Gastric cancer	GORD work up	HOP mass	Peri ampullary tumor	PSC
Actinomycetota	14,80	16,32	5,56	17,27	15,20	16,60	21,52	34,61	3,14
Bacillota	11,89	18,18	10,78	15,03	12,01	12,99	34,19	10,23	23,36
Bacteriodota	9,85	8,59	37,54	13,17	7,08	13,24	12,11	5,39	68,71
Chlamydiae	0	0	0,05	0	0	0	0	0	0
Cyanobacteria	0	0	0	0,03	0	0	0	0	0
Deferribacteres	0	0	0	0,04	0	0	0	0	0
Deinococcota	0,03	0,03	0	0,01	0	0	0,02	0,013	0
Fibrobacteres	0	0	0	0,01	0	0	0	0	0
Fusobacteria	4,80	0,76	17,28	1,13	0,223	7,12	1,52	0,49	1,02
Nitrospirae	0	0,01	0	0	0	0	0,01	0	0
Planctomycetes	0,03	0	0	0	0,10	0	0	0	0
Pseudomonadota	58,28	56,01	28,80	53,20	64,82	49,59	29,99	49,26	1,93
Spirochaetes	0,11	0,08	0	0,05	0,56	0,45	0,17	0	1,84
Synergistetes	0,09	0	0	0	0	0	0,01	0	0
Tenericutes	0,11	0,02	0	0,05	0	0	0,46	0	0
Verrucimicrobiota	0	0,01	0	0,01	0	0	0	0	0

Table S8. Impact of the multiple variables on the bacterial alpha diversity indices computed using Tukey HSD test.

Groups	Observed	Shannon
Cholelithiasis vs Choledocholithiasis	0,998	1,000
Distal Cholangiocarcinoma vs Choledocholithiasis	1,000	1,000
Epigastric pain vs Choledocholithiasis	1,000	0,998
Gastric Cancer vs Choledocholithiasis	1,000	1,000
GORD work up vs Choledocholithiasis	0,999	0,990
HOP mass vs Choledocholithiasis	1,000	1,000
Peri ampulary tumor vs Choledocholithiasis	0,999	1,000
PSC vs Choledocholithiasis	0,999	0,256
Distal Cholangiocarcinoma vs Cholelithiasis	1,000	1,000
Epigastric pain vs Cholelithiasis	1,000	0,989
Gastric Cancer vs Cholelithiasis	1,000	1,000
GORD work up vs Cholelithiasis	0,990	0,980
HOP mass vs Cholelithiasis	1,000	0,998
Peri ampulary tumor vs Cholelithiasis	1,000	1,000

PSC vs Cholelithiasis	1,000	0,317
Epigastric pain vs Distal Cholangiocarcinoma	1,000	0,994
Gastric Cancer vs Distal Cholangiocarcinoma	1,000	1,000
GORD work up vs Distal Cholangiocarcinoma	1,000	0,973
HOP mass vs Distal Cholangiocarcinoma	1,000	0,996
Peri ampulary tumor vs Distal Cholangiocarcinoma	1,000	0,999
PSC vs Distal Cholangiocarcinoma	1,000	0,858
Gastric Cancer vs Epigastric pain	1,000	0,997
GORD work up vs Epigastric pain	0,997	0,999
HOP mass vs Epigastric pain	1,000	1,000
Peri ampulary tumor vs Epigastric pain	1,000	1,000
PSC vs Epigastric pain	1,000	0,151
GORD work up vs Gastric Cancer	0,995	0,982
HOP mass vs Gastric Cancer	1,000	0,999
Peri ampulary tumor vs Gastric Cancer	1,000	0,999
PSC vs Gastric Cancer	1,000	0,824
HOP mass vs GORD work up	0,999	1,000
Peri ampulary tumor vs GORD work up	0,989	1,000
PSC vs GORD work up	0,991	0,248
Peri ampulary tumor vs HOP mass	1,000	1,000
PSC vs HOP mass	1,000	0,210
PSC vs Peri ampulary tumor	1,000	0,456

Table S9. Impact of the multiple variables on the bacterial alpha diversity indices within the duodenal mucosa samples obtained by endoscopic biospies, these differences were calculated using Dirichlet-Multinomial distribution test.

**Groups**	**chi-square**	**p-value**
Epigastric pain vs Cholelithiasis	1,30	0,99
Epigastric pain vs GORD work up	∞	0
Epigastric pain vs Choledocholithiasis	-18,65	1
Epigastric pain vs Distal Cholangiocarcinoma	∞	0
Epigastric pain vs Gastric cancer	∞	0
Epigastric pain vs HOP mass	8,90	0,35
Epigastric pain vs Peri ampulary tumor	∞	0
Epigastric pain vs PSC	∞	0
Cholelithiasis vs GORD workup	∞	0
Cholelithiasis vs Choledocholithiasis	3,84	0,87
Cholelithiasis vs Distal Cholangiocarcinoma	∞	0
Cholelithiasis vs Gastric cancer	∞	0
Cholelithiasis vs HOP mass	20,31	0,01
Cholelithiasis vs Peri ampulary tumor	∞	0
Cholelithiasis vs PSC	∞	0
GORD workup vs Choledocholithiasis	∞	0
GORD work up vs Distal Cholangiocarcinoma	∞	0
GORD workup vs Gastric cancer	∞	0
GORD workup vs HOP mass	∞	0

GORD workup vs Peri ampulary tumor	∞	0
GORD work up vs PSC	∞	0
Choledocholithiasis vs Distal Cholangiocarcinoma	∞	0
Choledocholithiasis vs Gastric cancer	∞	0
Choledocholithiasis vs HOP mass	1,16	0,1
Choledocholithiasis vs Peri ampulary tumor	∞	0
Choledocholithiasis vs PSC	∞	0
Distal Cholangiocarcinoma vs Gastric cancer	∞	0
Distal Cholangiocarcinoma vs HOP mass	∞	0
Distal Cholangiocarcinoma vs Peri ampulary tumor	∞	0
Distal Cholangiocarcinoma vs PSC	∞	0
Gastric cancer vs HOP mass	∞	0
Gastric cancer vs Peri ampulary tumor	∞	0
Gastric cancer vs PSC	∞	0
HOP mass vs Peri ampulary tumor	∞	0
HOP mass vs PSC	∞	0
Peri ampulary tumor vs PSC	∞	0
Stone disease vs non-stone disease	-7,19	1
Control vs Jaundice	10,65	0,22
Benign vs None	4,082	0,85
Malignant vs None	7,47	0,49
CLO test Positive vs Negative	-15,58	1
Gender Male vs Female	-2,59	1

"∞" represents no valid result
