# Supplementary figures and images for: Metataxonomic Analysis Demonstrates a Shift in Duodenal Microbiota in Patients with Obstructive Jaundice
Source: Microorganisms. 2023 Jun 18;11(6):1611. doi: 10.3390/microorganisms11061611 (PMC10301301; doi:10.3390/microorganisms11061611)

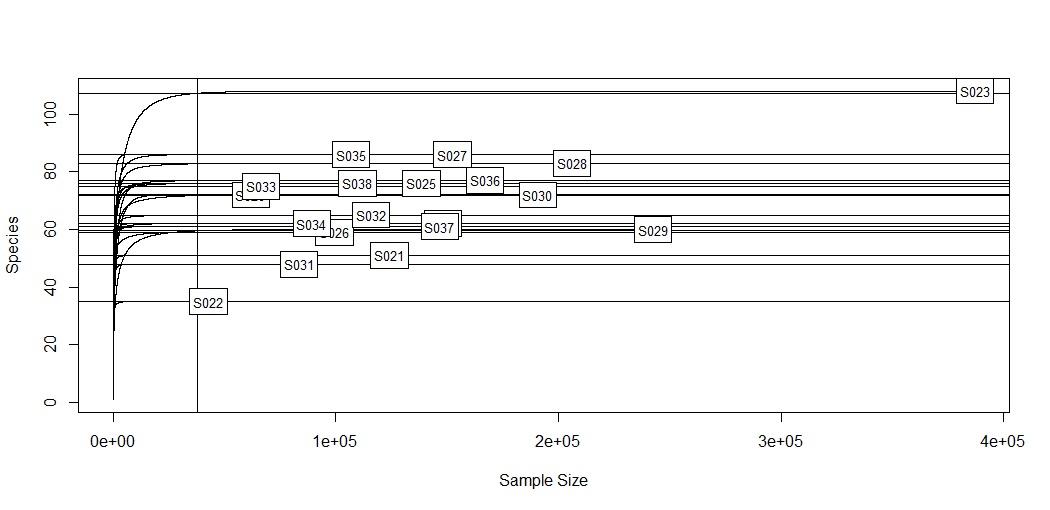

Supplement: Supplementary file 1 [file microorganisms-11-01611-s001.zip › Figure S1 (BOTTOM).jpg]

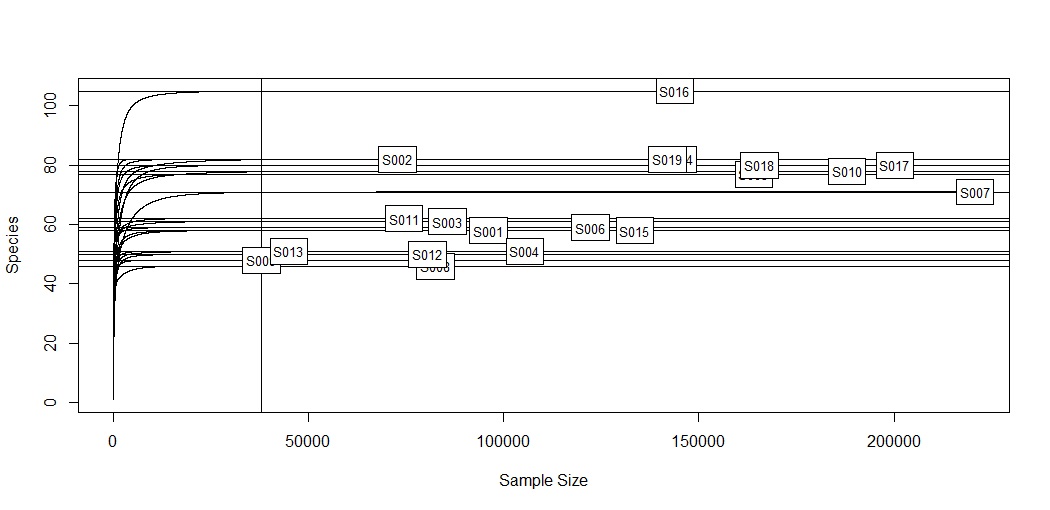

Supplement: Supplementary file 1 [file microorganisms-11-01611-s001.zip › Figure S1(TOP).jpg]

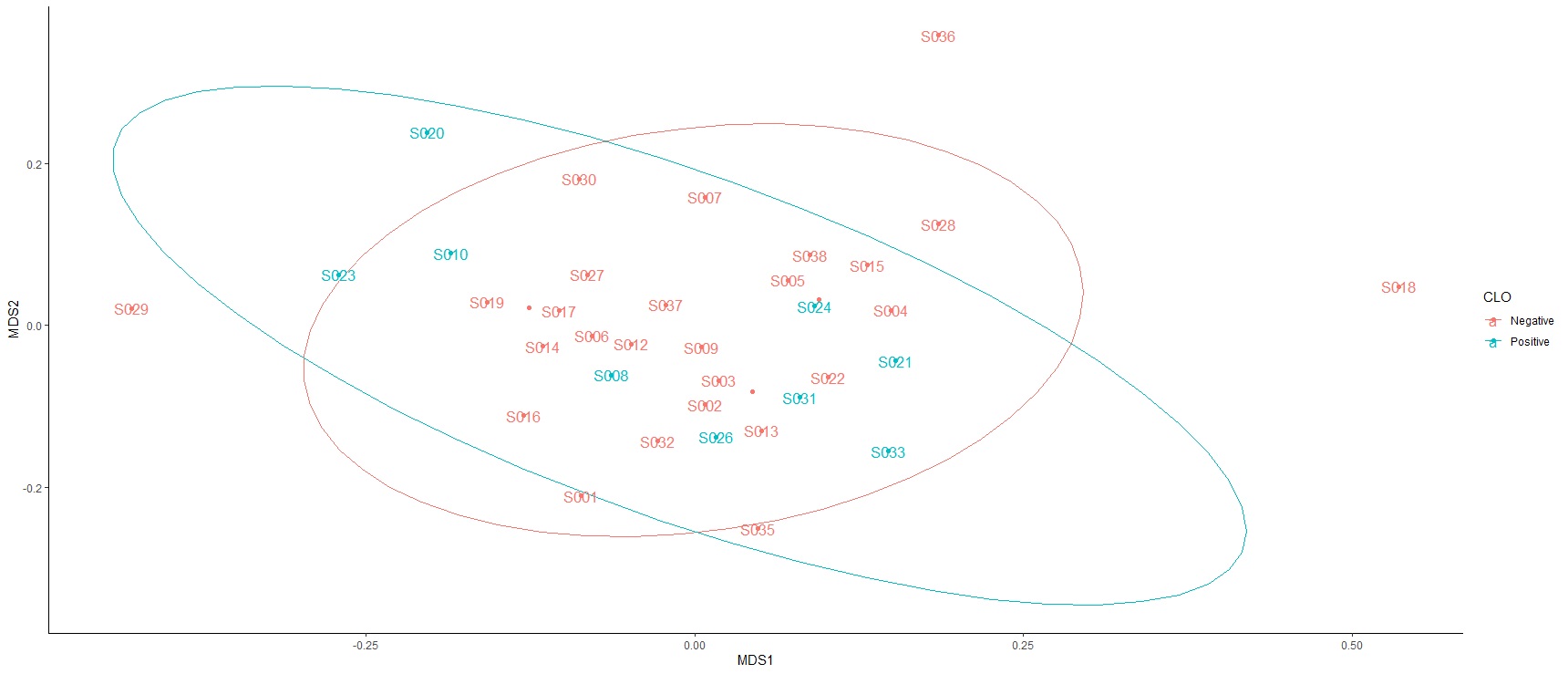

Supplement: Supplementary file 1 [file microorganisms-11-01611-s001.zip › Figure S2a.jpg]

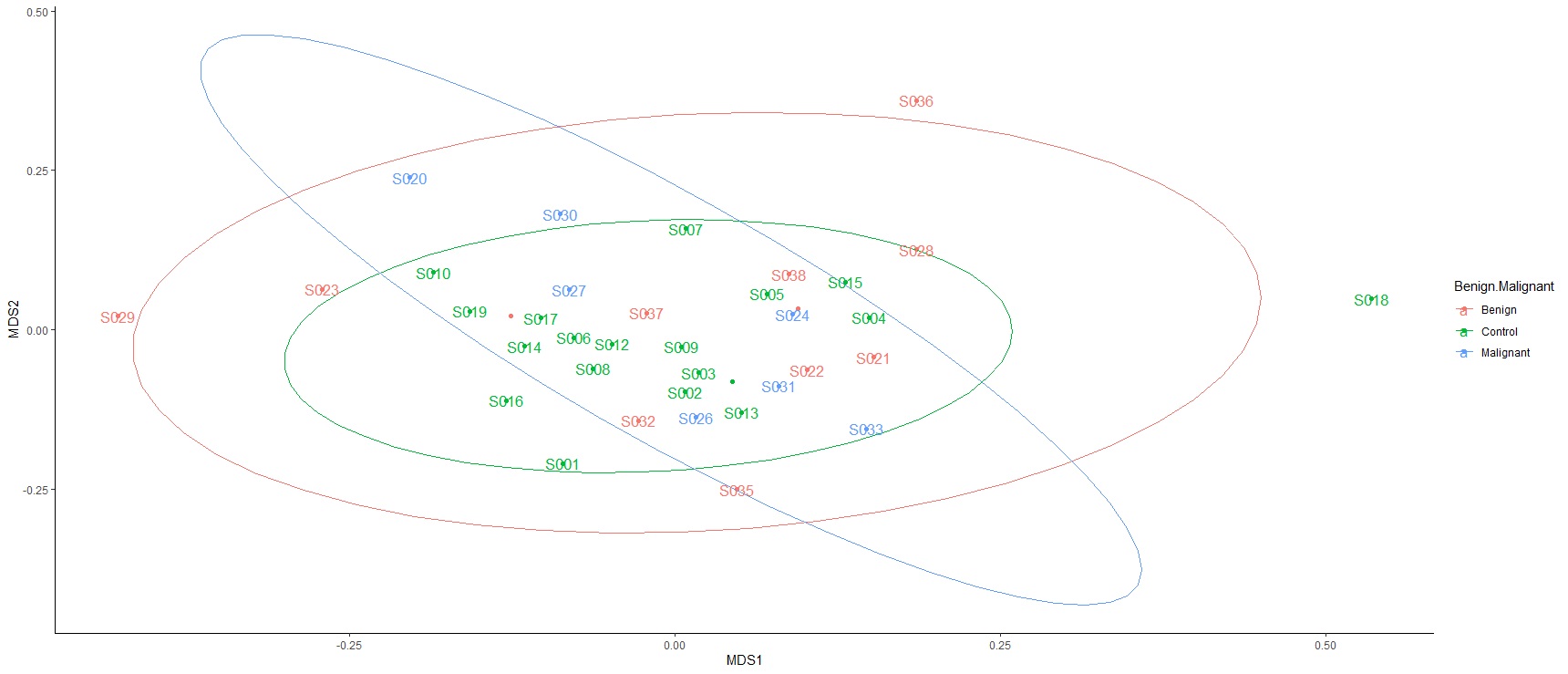

Supplement: Supplementary file 1 [file microorganisms-11-01611-s001.zip › Figure S2b.jpg]

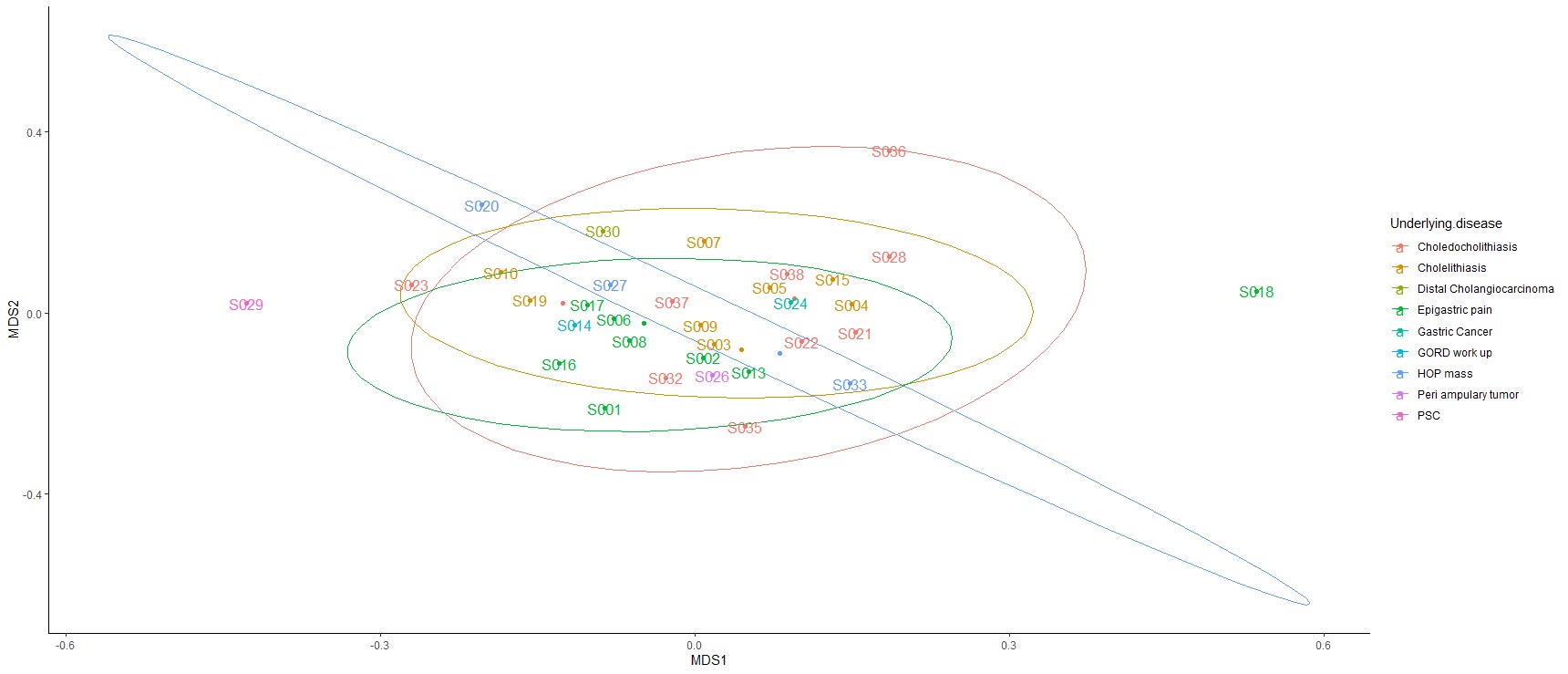

Supplement: Supplementary file 1 [file microorganisms-11-01611-s001.zip › Figure S2c.jpg]
